# The Nontoxic Cholera B Subunit Is a Potent Adjuvant for Intradermal DC-Targeted Vaccination

**DOI:** 10.3389/fimmu.2018.02212

**Published:** 2018-09-27

**Authors:** Laura Antonio-Herrera, Oscar Badillo-Godinez, Oscar Medina-Contreras, Araceli Tepale-Segura, Alberto García-Lozano, Lourdes Gutierrez-Xicotencatl, Gloria Soldevila, Fernando R. Esquivel-Guadarrama, Juliana Idoyaga, Laura C. Bonifaz

**Affiliations:** ^1^Hospital de Especialidades, Centro Médico Nacional Siglo XXI, Instituto Mexicano del Seguro Social, Unidad de Investigación Médica en Inmunoquímica, Mexico City, Mexico; ^2^Universidad Nacional Autónoma de México, Mexico City, Mexico; ^3^Centro de Investigación Sobre Enfermedades Infecciosas, Instituto Nacional de Salud Pública, SS, Cuernavaca, Mexico; ^4^Immunology and Proteomics Laboratory, Mexico Children's Hospital “Federico Gómez”, Mexico City, Mexico; ^5^Departamento de Inmunología, Instituto de Investigaciones Biomédicas, Universidad Nacional Autónoma de México, Mexico City, Mexico; ^6^Laboratorio de Inmunología Viral, Facultad de Medicina, UAEM, Cuernavaca, Mexico; ^7^Department of Microbiology and Immunology, Stanford University School of Medicine, Stanford, CA, United States

**Keywords:** anti-DEC205, CTB, adjuvant, skin, memory, T cells, dendritic cells

## Abstract

CD4^+^ T cells are major players in the immune response against several diseases; including AIDS, leishmaniasis, tuberculosis, influenza and cancer. Their activation has been successfully achieved by administering antigen coupled with antibodies, against DC-specific receptors in combination with adjuvants. Unfortunately, most of the adjuvants used so far in experimental models are unsuitable for human use. Therefore, human DC-targeted vaccination awaits the description of potent, yet nontoxic adjuvants. The nontoxic cholera B subunit (CTB) can be safely used in humans and it has the potential to activate CD4^+^ T cell responses. However, it remains unclear whether CTB can promote DC activation and can act as an adjuvant for DC-targeted antigens. Here, we evaluated the CTB's capacity to activate DCs and CD4^+^ T cell responses, and to generate long-lasting protective immunity. Intradermal (i.d.) administration of CTB promoted late and prolonged activation and accumulation of skin and lymphoid-resident DCs. When CTB was co-administered with anti-DEC205-OVA, it promoted CD4^+^ T cell expansion, differentiation, and infiltration to peripheral nonlymphoid tissues, i.e., the skin, lungs and intestine. Indeed, CTB promoted a polyfunctional CD4^+^ T cell response, including the priming of Th1 and Th17 cells, as well as resident memory T (RM) cell differentiation in peripheral nonlymphoid tissues. It is worth noting that CTB together with a DC-targeted antigen promoted local and systemic protection against experimental melanoma and murine rotavirus. We conclude that CTB administered i.d. can be used as an adjuvant to DC-targeted antigens for the induction of broad CD4^+^ T cell responses as well as for promoting long-lasting protective immunity.

## Introduction

Formulation of successful subunit vaccines requires the optimal combination of antigen and adjuvant to ensure the development of long-lasting protective immunity. Expected responses should include the development of memory CD4^+^ T cells, which play a major role in protecting against a myriad of pathogens (1–3) and against tumors (4, 5). To achieve this goal, delivering antigens via monoclonal antibodies (mAbs) targeting DCs, in combination with strong adjuvants, is one of the most promising strategies.

The administration of anti-DEC205-antigen mAbs can increase the efficiency of MHC-II antigen presentation relative to soluble antigen by 300-fold (4, 6). In combination with strong adjuvants, e.g., Poly IC, anti-CD40 mAbs, CpG, and flagellin (4, 7–9), it induces T helper (Th) cell differentiation and it mediates long-lasting immunity against experimental melanoma, malaria and influenza (4, 7, 10, 11). Moreover, DC-targeted vaccination can induce polyfunctional memory CD4^+^ T cells that produce IFNγ, TNFα and IL-2 (7, 9). Therefore, DC-targeted vaccination serves as a powerful strategy to promote protective CD4^+^ T cell responses.

Unfortunately, due to their toxicity, the adjuvants mentioned above are not approved for human use. Only synthetic derivatives, such as AS04 and phosphorothioate-backbone CpG adjuvants are undergoing trials with humans (12). However, these synthetic derivatives have shown adverse effects in murine models including splenomegaly, lymphoid follicle destruction and immunosuppression (12), which make them less promising for human use. Therefore, there is a need to identify adjuvants, which can be co-administered with DC-targeted antigens, for the induction of protective CD4^+^ T cell responses in humans.

CTB has been proven to be safe for human use as an adjuvant (13–15). Its use has been approved for the killed whole-cell monovalent vaccine (WC-rBS) against cholera in humans, which has only induced mild adverse effects in a few individuals, and it has been safe for and well-tolerated by immunocompromized subjects (16). Unfortunately, the capacity of CTB to activate DCs is controversial. Some *in vitro* studies using bone marrow-derived DCs (BMDCs) and macrophages (BMDM) show that CTB can promote expression of TLRs, CD86 and production of IL-5, IL-12p70, IL-6, IL-10, IL-3, G-CSF, MIP-2 and eotaxin, as well as it can activate the NFkB pathway (17, 18). In contrast, other studies suggest that CTB does not induce the activation of *ex vivo* DCs (19–21). Therefore, it is necessary to evaluate the capacity of CTB to activate DCs *in vivo*.

Still, several reports have shown that CTB can be used as a strong adjuvant. When admixed or conjugated with pathogen derived antigens, it can promote the generation of long-lived CD4^+^ T cells. Such responses mediate systemic immunity against several pathogens, including the influenza virus (22), *Helicobacter pylori* (23), *Streptococcus pneumoniae* (24), *Bordetella pertussis* (25), and *Francisella tularensis* (26). Furthermore, we have previously demonstrated that i.d. administration of soluble antigens in combination with CTB promotes CD4^+^ T cell activation and differentiation of Th1 and Th17 cells (27). However, CTB adjuvant's capacity has never been tested with DC-targeted antigens administered i.d. Here, we asked whether CTB co-administration with anti-DEC205-antigen mAbs could induce DC activation and consequently promote long-lasting and protective CD4^+^ T cell responses.

## Materials and methods

### Mice

WT C57BL/6 mice and transgenic mice expressing green fluorescent protein (GFP) under the major histocompatibility complex class II molecule promoter were obtained from Unidad de Medicina Experimental, UNAM animal facility. BALB/c mice were obtained from INSP, SS animal facility. OT-II CD45.1 mice were obtained from Instituto de Investigaciones Biomédicas, UNAM animal facility. All animal experiments were performed following the Institutional Ethics Committee and the Mexican national regulations on animal care and experimentation. Experiments with DO11.10 Thy1.1^+^ mice were performed at the Department of Microbiology and Immunology of the School of Medicine, at Stanford University, following institutional guidelines. Mice were sex (male or female)- and age (7–10 weeks)-matched.

### CD4^+^ T cell enrichment

Skin-draining lymph nodes (SDLN), spleen, and mesenteric lymph nodes were collected from OT-II CD45.1^+^ or DO11 Thy1.1^+^ mice, placed in RPMI medium (Gibco) supplemented with 5% fetal bovine serum (FBS) (HyClone), 300 μg/mL glutamine (Gibco) and 100 U/mL penicillin/100 μg/mL streptomycin (Biowest), and mashed separately to obtain cell suspensions. Red blood cells were lysed with RBC lysis buffer (Biolegend). Both LN and spleen suspensions were incubated for 30 min on ice with homemade rat hybridoma supernatants against CD8 (2.43), B cells (B220), MHCII-expressing cells (TIB120), and macrophages (F4/80). Next, cells were washed, suspended in supplemented RPMI and poured into petri dishes previously coated with rat anti-IgG (ThermoFisher) for 40 min at 4°C. Non-adherent cells were recovered, washed and suspended in PBS for injection through the retro orbital vein.

### Cell transfer and immunization

Congenic mice received 4.5–5 × 10^6^ CD4^+^ T cells intravenously (i.v.). After 24 h, anesthetized mice were immunized i.d. in both ears (or in the right flank for melanoma and viral challenge experiments) with 1 μg of anti-DEC205-OVA (containing ~0.5 μg of OVA protein), 1 μg of a control mAb-OVA without receptor affinity or 3–30 μg of soluble unconjugated OVA in the presence or absence of 10 μg of CTB (Sigma-Aldrich). For proliferation experiments mice received 4.5–5 × 10^6^ CFSE-labeled CD4^+^ T cells 24 h before i.d. administration of 1 μg of anti-DEC205-OVA or 1, 3, or 10 μg of soluble unconjugated OVA. For prime/boost experiments, mice were immunized i.d. in both ears with 1 μg of anti-DEC205-OVA or 3 μg of soluble unconjugated OVA plus 10 μg of CTB. After 15 days, mice received i.p. 1 μg of anti-DEC205-OVA or 3 μg of soluble unconjugated OVA.

### Tissue processing

At 3 or 7 days post-immunization, mice were sacrificed to collect SDLN and skin. SDLN were enzymatically digested with 0.25 mg/mL Liberase TL (Roche) and 0.125 mg/mL DNAse (Roche) for 25 min at 37°C. Skin cell suspensions were also obtained by enzymatic digestion with 0.25 mg/mL Liberase TL and 0.125 mg/mL DNAse for 45 min at 37°C, then chopped with scissors and incubated under the same conditions with constant shaking. Next, enzymatic digestion was stopped by adding 0.5 μM EDTA, and cell suspensions were filtered through a 70 μm strainer (Corning), followed by the addition of 0.125 mg/mL DNAse. Finally, cells were washed, counted, stained and/or re-stimulated as needed.

To obtain cells from the lungs, mice were sacrificed 7 days post-immunization. Lungs were rinsed with water to remove excess blood, placed into polypropylene tubes and chopped into small pieces to digest with 0.25 mg/mL Liberase TL (Roche) and 0.125 mg/mL DNAse (Roche) for 1 h at 37°C with constant shaking. Next, enzymatic digestion was stopped by adding 0.5 μM EDTA, and cell suspensions were filtered through a 70 μm strainer (Corning), followed by the addition of 0.125 mg/mL DNAse. Next, cells were lysed with the RBC lysis buffer (Biolegend). Finally, cells were washed, counted and stained.

Isolation of intestinal cells was performed as previously described elsewhere (28). Briefly: intestines were removed and carefully cleaned off their mesentery lymph nodes and Peyer's patches were excised. Intestines were opened longitudinally, washed off fecal contents, cut into pieces 0.5 cm in length, and subjected to two sequential 20-min incubations in HBSS with 5% FCS and 2 mM EDTA at 37°C with agitation to remove epithelial cells. After each incubation step, media containing epithelial cells and debris were discarded. The remaining tissue was minced and incubated for 20 min in HBSS with 5% FCS, 1 mg/ml collagenase IV and 40 U/ml DNase I at 37°C in agitation. Cell suspensions were collected and passed through a 100-μm strainer and pelleted by centrifugation at 300 g. Cells were counted and divided for *in vitro* re-stimulation and cell surface staining.

### *In vitro* re-stimulation

Cells were resuspended in RPMI medium supplemented with 10% FBS, 300 μg/mL glutamine, 100 U/mL penicillin/100 μg/mL streptomycin, 110 μg/mL sodium pyruvate and 10 μM β-mercaptoethanol. SDLN cells were incubated for 48 h with OVA peptide 323–339 (in vivogen), followed by cell stimulation cocktail plus protein transport inhibitor, added according to the manufacturer's instructions (eBioscience), and cells were incubated for an additional 4 h at 37°C. Cells from the skin and intestine were only re-stimulated with cell cocktail stimulation plus protein transport inhibitor for 4 h without OVA.

### Flow cytometry

To allow for counting, cells were stained with anti-CD45-PECy7 (Biolegend) and DAPI (ThermoFisher), immediately mixed with CountBright absolute counting beads (ThermoFisher), acquired for flow cytometry. Cell surface staining was performed first by blocking Fc receptors (supernatant of 2.4G2 hybridoma against CD16/32) and then by staining using the following antibodies: anti-CD45-APC (Biolegend) or -PECy7 (Biolegend), anti-CD4-APC-Cy7 (Biolegend), anti-TCRVβ5.1, 5.2-PECy7 (Biolegend) or anti-Vα2-FITC (eBioscience), anti-CD45.2-Percp-Cy5.5 (Biolegend) or anti-CD45.1-Percp-Cy5.5 (Biolegend), anti-CD69-PE (ebioscience), and anti-CCR7-FITC (Biolegend). LIVE/DEAD Fixable Aqua (Thermofisher) staining was included. For DC analysis the following Abs were used: anti-CD45-APC (Biolegend), anti-Ter119-Percp-Cy5.5, anti-CD3-Percp-Cy5.5, anti-CD19-PercpCy5.5, anti-CD44b-Percp-Cy5.5, anti-MHCII-FITC (Biologend), and CD86-PE (eBioscience). To achieve intracellular staining, cell surface staining was first performed, followed by fixation and permeabilization using the intracellular fixation and permeabilization buffer set (Thermofisher), according to the manufacturer's instructions. To stain cytokine and transcription factors, the True-Nuclear transcription factor buffer set (Biolegend) was used according to the manufacturer's instructions. Intracellular staining included anti-IL-17-PE (BD Bioscience), anti-IFNγ-APC (Biolegend), anti-T-bet-BV421 (BD Biosciences), or anti-RORγT-APC (Thermofisher). Cells were acquired in a BD FACSCanto II or BD LSRFortessa cytometer (Becton, Dickinson and company). Data were analyzed with FlowJo software (Tree Star, Inc.).

### Melanoma challenge

Mice were transferred with OT-II CD45.1^+^ cells 24 h before i.d. immunization with 1 μg of anti-DEC205-OVA or with 3 μg of soluble untargeted OVA ± 10 μg of CTB. After 30 days, mice received 2.5 × 10^5^ MO4 cells subcutaneously (s.c.) in the right flank and then they were monitored for 21 days for survival. Alternatively, C57BL/6 naive mice were challenged i.v. in the tail vein 30 days after immunization to induce metastatic nodules in the lungs. For some experiments, anti-DEC205-OVA-vaccinated mice received i.p. 250 μg of anti-CD4 Ab (GK1.5, in house) or isotype control Ab (eBRG1, in house) as follows: 1 day before MO4 inoculation, on the day of MO4 inoculation and every 3 days after MO4 inoculation, up to day 12. Sixteen days after MO4 inoculation, mice were sacrificed and lungs were harvested for metastatic nodule count as described elsewhere (29). Briefly: lungs were rinsed with water to remove excess blood and bleached with Feket's solution, and metastatic nodules were counted under a stereoscope (Leica Microsystems). Uncountable nodules were reported as >250.

### Viral challenge

BALB/c mice were immunized i.d. in the right flank with 23 μg of anti-DEC205-VP6 (corresponding to 1.5 μg of VP6) or with 3 μg of *in vitro* synthetized soluble untargeted VP6 (produced from the murine rotavirus Ew *in vitro* with the Rapid Translation System, Roche), in the presence of 10 μg of CTB. After 20 days, mice were orally challenged with 1 × 10^4^ focus forming units of murine RV EDIM_WT_ as described elsewhere (30). For prime/boost experiments, mice were i.d. immunized with anti-DEC205-VP6 or 3 μg of VP6 plus 10 μg of CTB and, after 15 days, mice received i.p. anti-DEC205-VP6 or VP6 (same dose as before). For CD4^+^ T cell depletion experiments, mice immunized with anti-DEC205-VP6 received either 250 μg of anti-CD4 Ab (GK1.5, in house) or isotype control Ab (eBRG1, in house) as follows: 3 days before the viral challenge, on the day of the challenge and 3 days after the challenge. Seven days after boost, mice were orally challenged with 1 × 10^4^ focus forming units of murine RV EDIM_WT_. Stool samples were collected daily for 8 days and kept at −20°C for further analysis of viral load by sandwich ELISA. Protection against infection was calculated as % protection = 100% – [area under the curve of the experimental group (Absorbance at 405 nm)/area under the curve of the control group (Absorbance at 405 nm)] × 100%. This represents a decrease in the quantity of rotavirus antigen shed after immunization, relative to control mice, during the 8 days after the challenge.

### ELISA

Viral load in the stool was determined by sandwich ELISA, as described elsewhere (30). Briefly: diluted stool samples were poured into 96-well plates (Costar) previously coated with a goat polyclonal antibody (Ab) against different strains of RV (in house). After 2 h at 37°C, plates were washed, and a rabbit polyclonal Ab against RV RRV was added. After 1 h at 37°C, plates were washed and a PA-conjugated goat anti-rabbit IgG (Zymed) was added, which was incubated for 1 h at 37°C. Finally, after washing, the substrate (*p*-nitrophenyl phosphate, disodium; Sigma) was added, and plates were developed for 30–45 min at 37°C. The absorbance at 405 nm was read with a 96-well plate reader (BIO-TEK Instruments, Burlington, VT).

### DC activation

GFP-MHC-II mice received 10 μg of CTB or PBS i.d. in the ear. After 12, 24 or 72 h, epidermal sheets were obtained, stained with anti-CD86-PE (eBioscience), mounted with VectaShield (Vector Laboratories) and sealed. The images were obtained with a Leica TCS SP8x Confocal Microscope (Wetzlar, Germany) and analyzed with Leica Application Suite Advanced Fluorescent Lite software (Leica Microsystems, Mannheim, Germany). Alternatively, C57BL/6 mice received 10 μg of CTB or PBS i.d. in the ear. After 24, 72 h, or 7 days, mice were sacrificed to collect SDLN and skin. Tissues were processed and stained to be analyzed by flow cytometry.

### Statistics

Statistical analysis was performed using Prism 6.0 (GraphPad Software Inc., La Jolla, CA, USA). Statistical significance was calculated when comparing two groups, using unpaired two-tailed Student's *t*-test. For comparison of more than two groups, one-way or two-way ANOVA with Tukey's multiple comparison test was used. A *P-*value < 0.05 was considered significant.

## Results

### CTB induces late and prolonged activation and accumulation of SDLN and skin DCs

**Figure 1:**
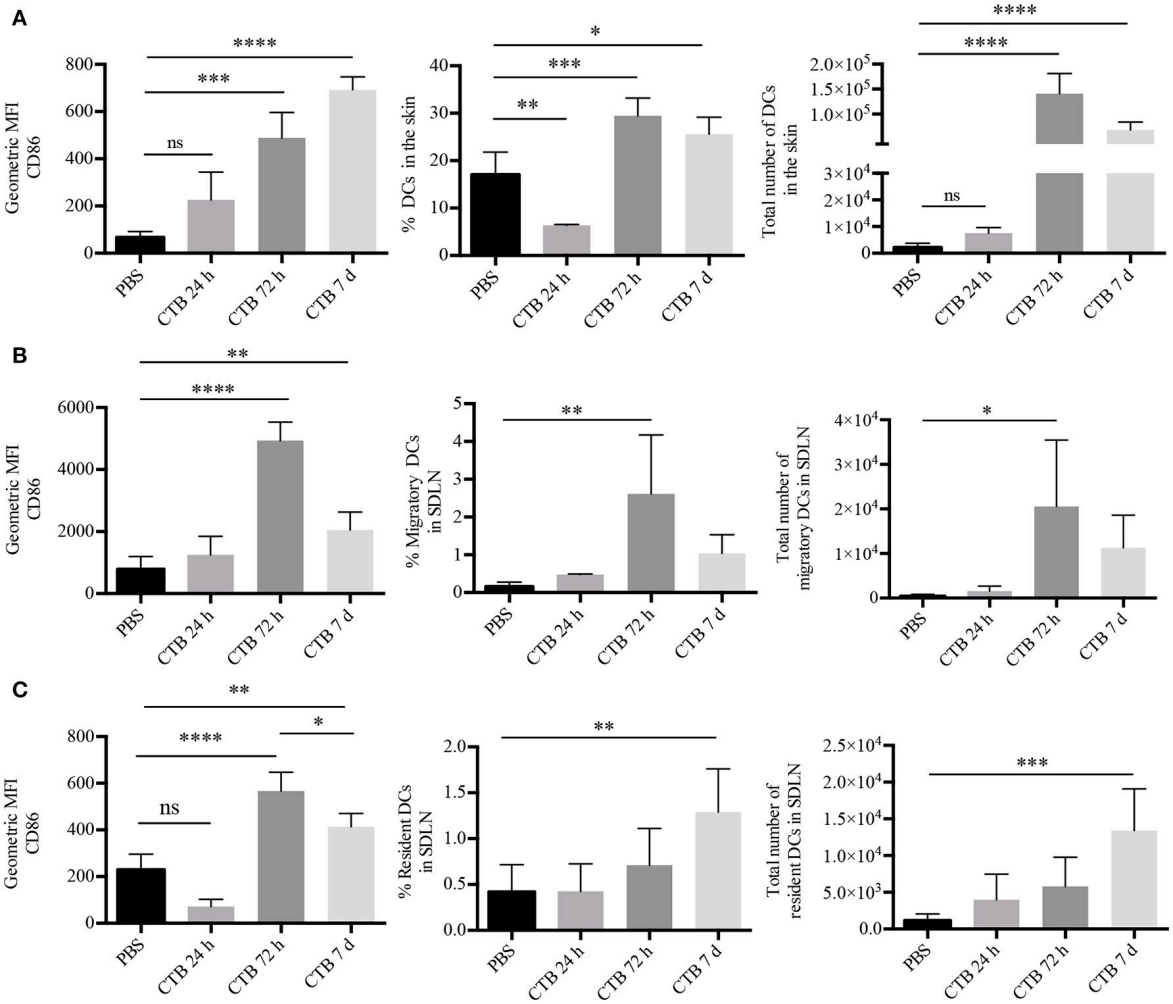
Intradermal administration of CTB promotes recruitment and activation of DCs in the SDLN and the skin. C57BL6 mice received 10 μg of CTB or PBS i.d. in both ears, and they were sacrificed for skin and SDLN harvesting at the indicated times. **(A)** MHC-II^+^CD11c^+^ DCs were gated as in Supplementary Figure 2A. Graphs depicting the percentage, absolute cell numbers of DCs and geometric median fluorescence intensity (MFI) of CD86 on DCs in the skin. Mean ± SD, *N* = 4–6, data pooled from two independent experiments. One-way ANOVA with Tukey's multiple comparisons test (*P < 0.05, **P < 0.005, ***P < 0.0005, ****P < 0.0001). **(B)** Migratory and **(C)** resident DCs from the SDLN were gated as in Supplementary Figure 2B. Graphs of percentage, total numbers of DCs and geometric MFI of CD86 on DCs. Mean ± SD, *N* = 4–6, data pooled from two independent experiments. One-way ANOVA with Tukey's multiple comparisons test (*P < 0.05, **P < 0.005, ***P < 0.0005, ****P < 0.0001).

We first aimed to determine whether CTB could induce *in vivo* activation of DCs. To this end, epidermal sheets of GFP-MHC-II mice were obtained at 12, 24, or 72 h after i.d. administration of CTB; followed by staining with fluorescent Ab specific for CD86. Using confocal microscopy, we observed co-expression of CD86 by epidermal MHC-II^+^ cells, only after 72 h, and at no earlier time (Supplementary Figure 1). Next, we characterized skin DCs as viable CD45^+^Lineage^−^CD11c^+^MHC-II^+^ cells by multiparametric flow cytometry (Supplementary Figure 2A). We confirmed that CTB induces *in vivo* activation of DCs after 72 h by overexpression of CD86 (Figure 1A) and, interestingly, their accumulation in the inoculation site as well. It was striking that both the activation and accumulation were sustained 7 days after the i.d. administration of CTB (Figure 1A).

Next, we asked whether CTB could induce accumulation of activated DCs in the SDLN. To answer this question, we analyzed SDLN cells by multiparametric flow cytometry, which allowed us to discriminate between migrating (CD11c^+^MHC-II^hi^) and resident (CD11c^+^MHC-II^low^) DCs (Supplementary Figure 2B). Seventy-two hours after its administration, CTB induced the accumulation of migratory DCs in the SDLN, which displayed an increased expression of CD86 compared to the PBS control (Figure 1B). The accumulation of migrating DCs with an activated phenotype dropped after 72 h. However, it was still higher than the PBS control after 7 days. Interestingly, CTB also induced an increased expression of CD86 on resident DCs as well as their accumulation after 7 days (Figure 1C). It is worth noting that the accumulation and activation of DCs took place only at the inoculation site and the draining lymph node, as we did not find either effect on a distal organ, i.e., the mesenteric lymph nodes (Supplementary Figure 2C).

As a whole, our results demonstrate that skin administration of CTB acts as a potent stimulus to induce late and prolonged accumulation and activation of lymphoid-resident and skin DCs.

### CTB co-administration with a DC-targeted or soluble antigen promotes expansion and differential activation of CD4^+^ T cells

**Figure 2:**
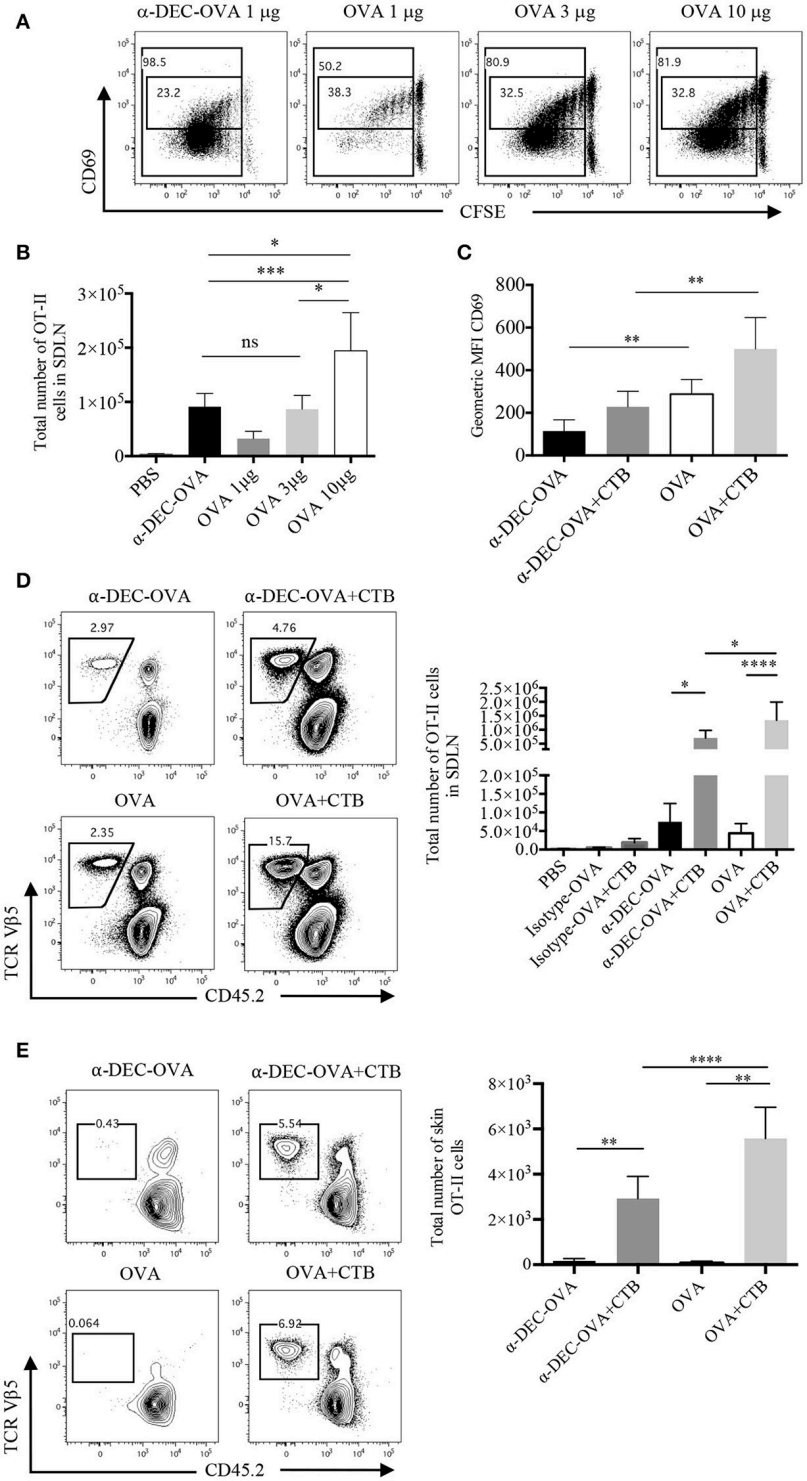
CTB co-administration with a DC-targeted or soluble antigen promotes expansion and differential activation of CD4^+^ T cells. C57BL6 mice were adoptively transferred with OT-II CD45.1^+^ cells, 24 h later they were immunized i.d. in both ears, as indicated, and 3 or 7 days later, they were sacrificed for SDLN and skin harvesting. **(A)** Representative dot plot of CFSE dilution and CD69 expression by SDLN OT-II cells 3 days after inoculation of anti-DEC205-OVA or soluble OVA and **(B)** total numbers of OT-II cells. **(C)** Geometric median fluorescence intensity (MFI) of CD69 by OT-II cells 3 days after anti-DEC205-OVA or soluble OVA ± CTB's i.d. administration. Mean ± SD, *N* = 4–6, data pooled from four independent experiments. One-way ANOVA with Turkey's multiple comparisons test (ns, *P* > 0.05, *P < 0.05, **P < 0.005, ***P < 0.0005). **(D)** Representative dot plots and total number of SDLN OT-II cells 7 days after anti-DEC205-OVA or soluble OVA ± CTB's i.d. administration. Mean ± SD, *N* = 5–8 data pooled from four independent experiments. One-way ANOVA with Tukey's multiple comparisons test (*P < 0.05, ****P < 0.0001). Transferred cells recovered from the SDLN were identified as viable CD4^+^CD45.2^−^TCRVβ 5.1, 5.2^+^ T cells (Supplementary Figure 3A). **(E)** Representative dot plot and total numbers of migrating OT-II cells identified as viable CD45^+^CD4^+^CD45.2^−^TCRVβ 5.1, 5.2^+^ (Supplementary Figure 3C). Mean ± SD, *N* = 4–6, data pooled from four independent experiments. One-way ANOVA with Tukey's multiple comparisons test (**P < 0.005, ****P < 0.0001).

To study the development of antigen specific CD4^+^ T cell responses we used a DC-targeted OVA antigen and, for comparison, soluble OVA antigen. After 3 days, we observed a 20-fold increase in the proliferation of CD4^+^ T cells after the i.d. inoculation of 1 μg of anti-DEC205-OVA, compared to 10 μg of soluble OVA (Supplementary Figure 3B). Furthermore, cells undergoing the last rounds of proliferation showed downregulation of CD69, which was more pronounced in cells from anti-DEC205-OVA-inoculated mice (Figure 2A). CD69 is rapidly activated after TCR engagement, but it decreases as T cells divide (31, 32). Even so, similar numbers of OVA-specific CD4^+^ T cells were found in the SDLN of mice administered with 3 μg of soluble OVA or with 1 μg of anti-DEC205-OVA (Figure 2B).

Next, we evaluated the outcome of CTB co-administration in T cell activation. Three days post-immunization, cells from mice administered with CTB plus a DC-targeted antigen remained low for CD69 expression; while a soluble antigen admixed with CTB resulted in higher expression of CD69 (Figure 2C). The expression of CD69 promotes retention of T cells in the lymph node; while its deregulation allows cells to migrate to distal peripheral tissues (31, 32). Thus, similar to others (6, 33), our data suggest the possibility of systemic dissemination of CD4^+^ T cells after DC-targeted antigen inoculation.

After 7 days, we observed a significant effect on T cell expansion, as CTB co-administered with a DC-targeted antigen promoted larger numbers of OVA-specific CD4^+^ T cells (Figure 2D). This result was dependent on the antigen being targeted to DCs, since the administration of the isotype Ab conjugated with OVA, with or without CTB, did not promote expansion (Figure 2D; Supplementary Figure 3B). CTB co-administration with soluble OVA promoted larger accumulation of CD4^+^ T cells in the SDLN as compared with the DC-targeted OVA group (Figure 2D), and it was consistent with a higher expression of CD69.

We next asked if CTB could promote the migration of antigen-specific CD4^+^ T cells to the inoculation site. After 7 days of i.d. immunization, we observed a large infiltration of OT-II CD45.1^+^ cells in the skin, which was promoted by the co-administration of CTB and not by the antigen alone (Figure 2E). Strikingly, higher numbers of OVA-specific T cells were observed in the skin of mice immunized with soluble OVA along with CTB compared to the DC-targeted vaccination group.

All together, these data demonstrate that CTB can be used as a strong adjuvant with a DC-targeted or soluble antigen to promote local expansion of antigen-specific CD4^+^ T cells in the SDLN, and to induce their efficient migration to the inoculation site (i.e., skin). Remarkably, our data suggest that a DC-targeted antigen induces differential activation of CD4^+^ T cells, which might impact their differentiation and, possibly, the differential anatomical localization of CD4^+^ T cells after DC-targeted or soluble antigen immunization.

### CTB promotes a combined Th1/Th17 response when co-administered with a DC-targeted antigen

**Figure 3:**
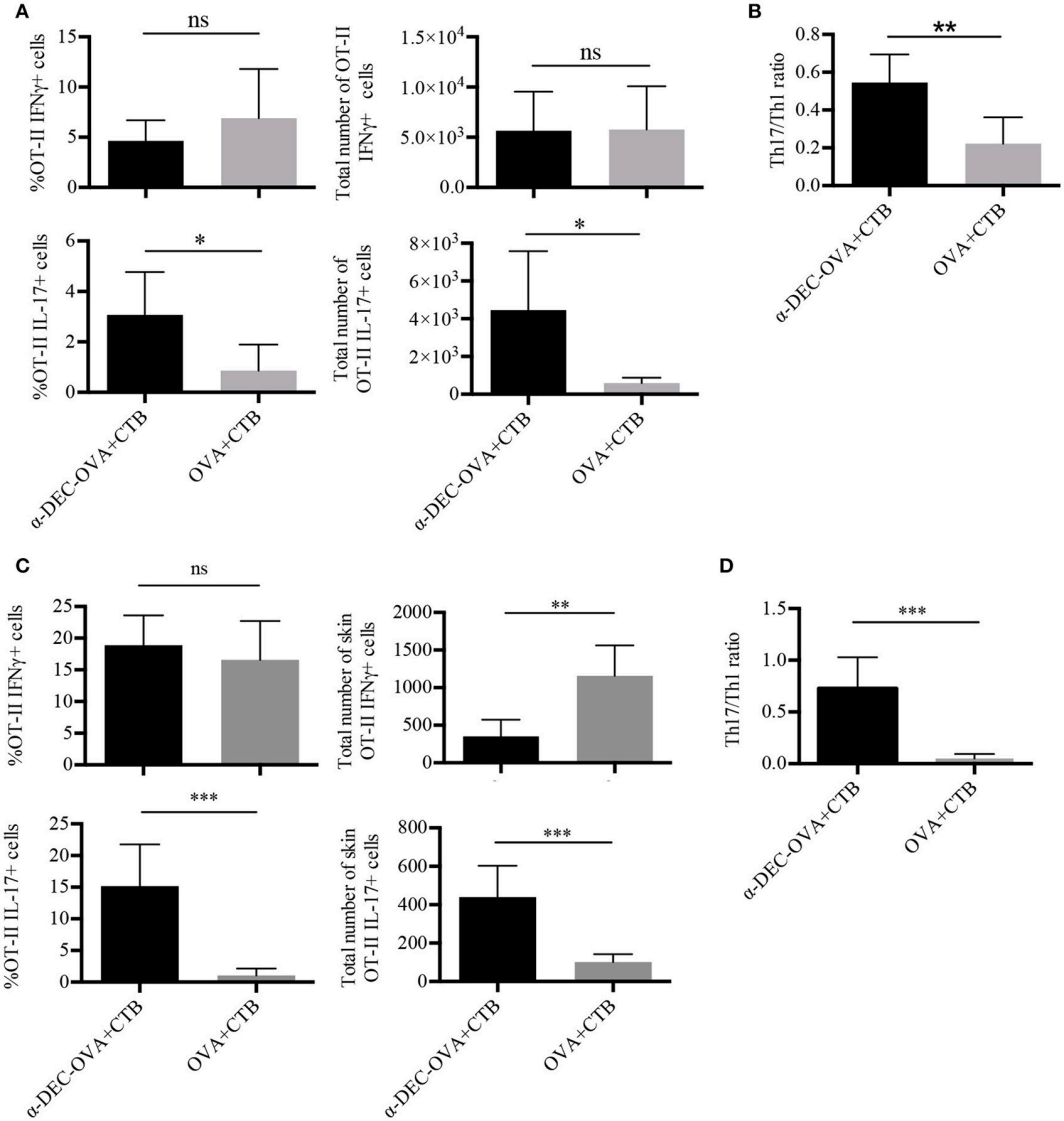
CTB promotes Th1 and Th17 differentiation and recruitment to the skin after i.d. co-administration with a DC-targeted antigen. Mice were treated as in Figure 2, and 7 days after immunization, the SDLN and skin were collected to obtain cell suspensions for *in vitro* re-stimulation. **(A)** Cells from the SDLN were incubated for 48 h with OVA 323–339 peptide followed by 4 h with cell cocktail stimulation + protein transport inhibitor. Graphs of percentage and total numbers of IFNγ^+^ and IL-17^+^ OT-II cells (identified as in Figure 2A). **(B)** Ratio of SDLN Th17/Th1 cells. Mean ± SD, *N* = 6–8, data pooled from two independent experiments. Unpaired *T*-test (ns, *P* > 0.05, *P < 0.05, **P < 0.005). Skin cell suspensions were stimulated with cell cocktail stimulation + protein transport inhibitor for 4 h. **(C)** Graphs of percentage and total numbers of skin IFNγ^+^ and IL-17^+^ of OT-II cells (identified as in Figure 2B). **(D)** Ratio of skin Th17/Th1 cells. Mean ± SD, *N* = 6–8, data pooled from three independent experiments. Unpaired *T*-test (ns, *P* > 0.05, **P < 0.005, ***P = 0.0001).

We next asked whether CTB admixed with a DC-targeted antigen or a soluble antigen could promote the differentiation of CD4^+^ T cells into Th1 or Th17 cells. At day 7 post-immunization, we observed antigen-specific IFNγ^+^ cells in the SDLN, induced by the administration of CTB in combination with a DC-targeted antigen or a soluble antigen (Supplementary Figure 4A; Figure 3A). Remarkably, only DC-targeted vaccination promoted significant differentiation of IL-17^+^ CD4^+^ T cells (Supplementary Figure 4A; Figure 3A). These results were confirmed in the DO11.10 model (Supplementary Figure 5A), which is prone to Th2 and Treg responses. Moreover, IL-17^+^ and IFNγ^+^ cells expressed the transcription factors RORγt and T-bet, respectively (Supplementary Figure 4A). Thus, DC-targeted vaccination promoted a combined Th1/Th17 response in the SDLN, in contrast to soluble antigen, which induced mainly Th1 responses (Figure 3B).

We then analyzed skin-infiltrating T cells. Immunization with either DC-targeted OVA or soluble OVA together with CTB induced a similar percentage of Th1 CD4^+^ T cells (Figure 3C). However, DC-targeted OVA + CTB induced a higher frequency of and absolute cell numbers of Th17, compared to soluble OVA + CTB (Supplementary Figure 4B; Figure 3C). Indeed, we confirmed that DC-targeted OVA + CTB promote a combined Th1/Th17 response in the skin, while immunization with the soluble OVA + CTB promotes a skewed Th1 response by calculating the Th1/Th17 ratio (Figure 3D). Similarly, we also observed great infiltration of Th17 cells and almost no Foxp3^+^ regulatory T cell differentiation in the skin of BALB/c mice transferred with DO11.10 cells after DC-targeted OVA + CTB administration (Supplementary Figures 5B,C).

As a whole, our results demonstrate that CTB, in combination with a DC-targeted antigen, promotes a combined Th1 and Th17 response, while soluble antigen vaccination promotes a skewed Th1 response.

### Antigen targeting to DCs along with CTB promotes CD4^+^ T RM cell differentiation in the skin

**Figure 4:**
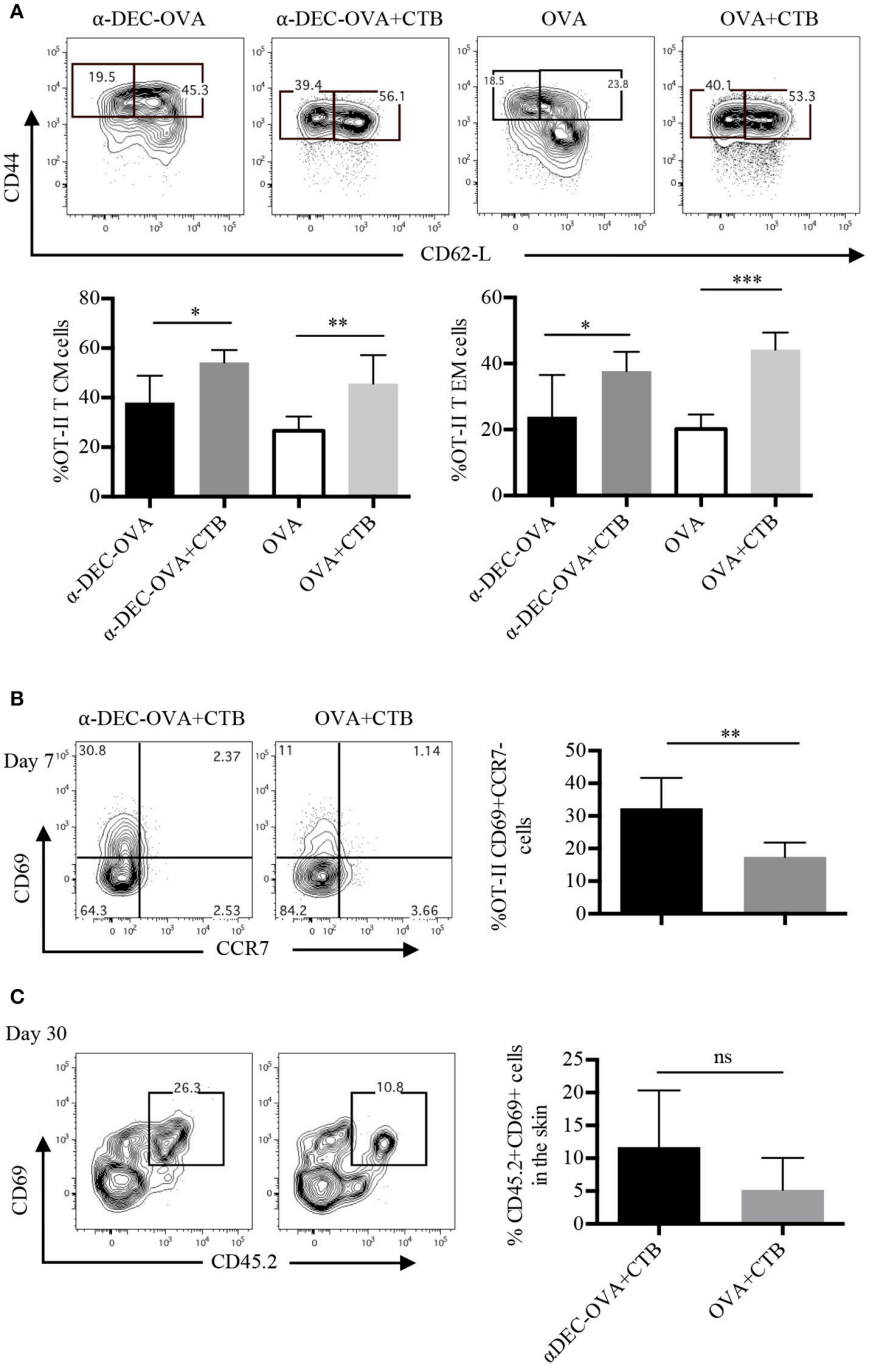
Antigen targeting to DCs along with CTB promotes T EM, T CM and T RM cell differentiation. Mice were treated as in Figure 2 and the SDLN along with the ears were collected at the indicated times. **(A)** Representative contour plots of T EM (CD44^+^CD62L^−^) and of T CM (CD44^+^CD62L^+^) cells from OT-II CD45.1^+^ cells (identified as in Figure 2A), and graphs of the percentage of each population 7 days post-immunization. Mean ± SD, *N* = 4–6, data pooled from two independent experiments. One-way ANOVA with Tukey's multiple comparisons test (*P < 0.05, **P < 0.005, ***P < 0.0005). **(B)** Representative contour plots and a graph showing percentages of CD69^+^CCR7^−^ OT-II CD45.1^+^ cells (identified as in Figure 2B) from the inoculation site 7 days post-immunization. Mean ± SD, *N* = 5–6, data pooled from three independent experiments. **(C)** CD45.1^+^ mice received i.v. OT-II CD45.2^+^ cells and 1 day later were inoculated with 1 μg of anti-DEC205-OVA or with 30 μg of OVA, both in combination with CTB. Representative contour plots and a graph showing percentages of CD69^+^ OT-II CD45.2^+^ cells 30 days post-immunization. Mean ± SD, *N* = 3–5 data pooled from two independent experiments. Unpaired *T*-test.

We next aimed to dissect the memory response induced by a DC-targeted antigen or a soluble antigen in combination with CTB. We first characterized the circulating and re-circulating memory of the CD4^+^ T cell pool in the SDLN of immunized mice. CD4^+^ T cells were classified as central memory (T CM) T cells or effector memory (T EM) T cells, according to their expression of CD44 and CD62L. The CTB's co-administration promoted increased differentiation of both T CM and T EM antigen-specific CD4^+^ T cells in the SDLN, in combination with a DC-targeted or soluble antigen (Figure 4A).

Next, we studied the differentiation of skin-resident memory CD4^+^ T cells [T RM; CD69^+^CCR7^−^ (34)] after immunization. At the effector stage, a fraction of T cells that migrate to nonlymphoid organs acquire the expression of CD69 just upon their arrival to these sites (35), which can give rise to a smaller population of long-lived T RM cells (36). Accordingly, 7 days post i.d. immunization, we found that ~30% of OT-II cells were CD69^+^CCR7^−^ cells after DC-targeted OVA + CTB and, surprisingly, only ~15% after soluble OVA + CTB immunization (Figure 4B). Furthermore, 30 days post-immunization, most of the OVA-specific CD4^+^ T cells from the skin of DC-targeted OVA + CTB mice were CD69^+^ (Figure 4C). Interestingly, a DC-targeted antigen was more efficient at generating long-lived T RM cells, even in comparison with a high dose of soluble OVA (30 μg of OVA, which is ~60 times more than the amount of OVA contained in 1 μg of anti-DEC205-OVA; Figure 4C).

All together, our findings show that CTB can be used to enhance the differentiation of central and effector memory CD4^+^ T cells, and that its combination with an antigen targeted to DCs efficiently promotes the differentiation of skin CD4^+^ T RM cells.

### Intradermal immunization with CTB along with a DC-targeted antigen provides local and systemic long-lasting immunity

**Figure 5:**
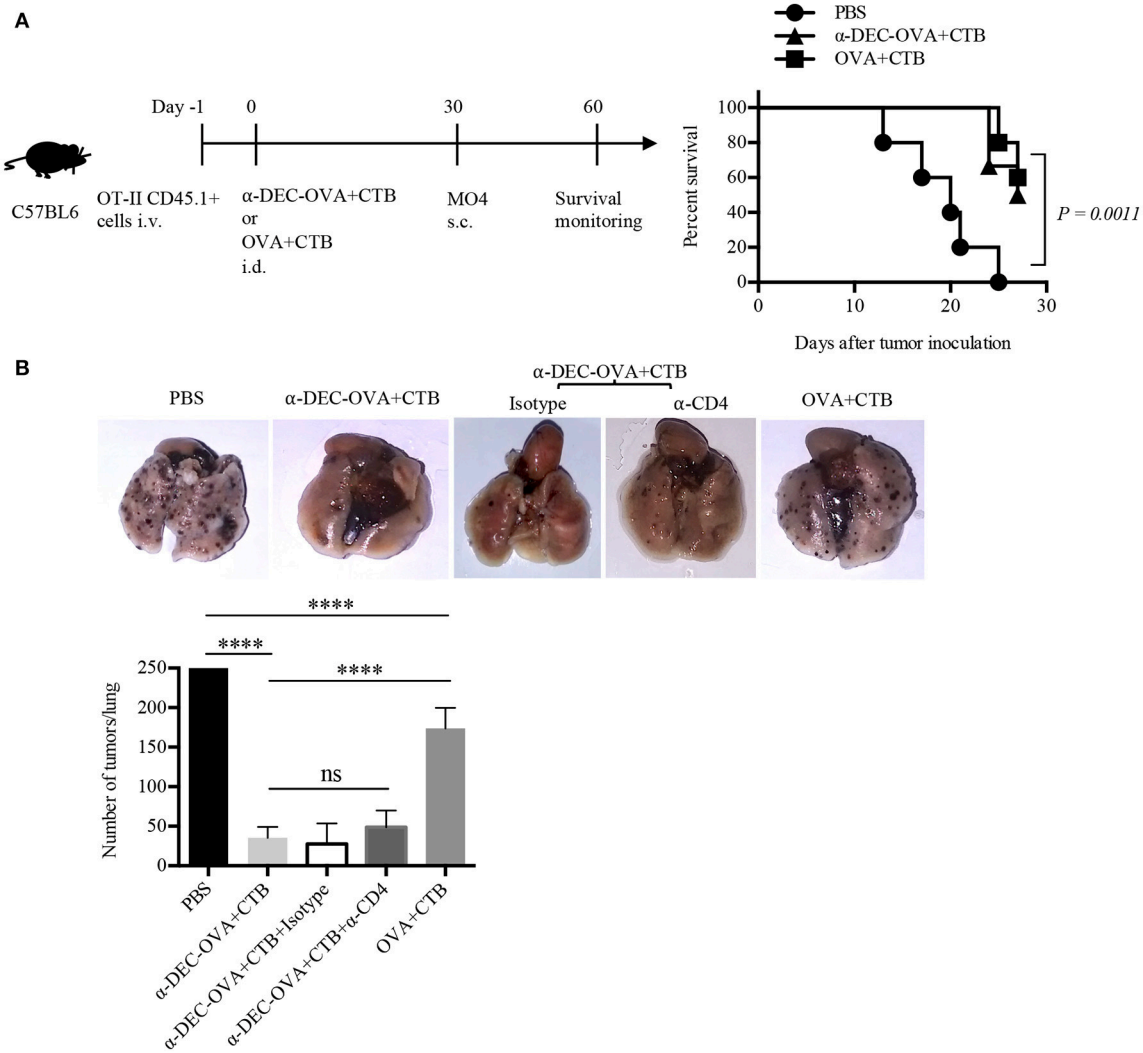
Intradermal immunization with CTB, along with a DC-targeted antigen, provides local and systemic long-lasting immunity against melanoma. **(A)** Diagram showing the strategy followed for immunizations and a graph showing survival rate after MO4 s.c. challenge. *N* = 5 per group, data pooled from two independent experiments. Log-rank (Mantel-Cox) test. Naïve mice were i.d. immunized as indicated, and after 30 days i.v. challenged with MO4 cells. Mice immunized with anti-DEC-OVA+CTB received i.p. anti-CD4 or the control isotype Ab, before, during, and after the inoculation of MO4 cells. **(B)** Representative pictures of lungs and a graph of metastatic nodules per lung, 16 days after challenge. Mean ± SD, *N* = 5–10, data pooled from two independent experiments. One-way ANOVA with Tukey's multiple comparisons test (ns, *P* > 0.05, ****P < 0.0001).

The fact that the CTB's i.d. co-administration with a DC-targeted antigen promoted CD4^+^ T cell activation, Th1/Th17 differentiation and migration to the skin, as well as CD4^+^ TRM cell differentiation, prompted us to investigate whether this immunization strategy could translate into protective long-term immunity. Thus, we first made use of the subcutaneous OVA-expressing melanoma model (Figure 5A). We found that i.d. immunization with DC-targeted OVA or soluble OVA in combination with CTB promoted local protection against a subcutaneous challenge with an OVA-expressing melanoma (Figure 5A).

To evaluate if the CTB's co-administration with a DC-targeted antigen could elicit systemic activation of T cells, mice vaccinated i.d. were i.v. challenged with MO4 cells. Mice immunized with DC-targeted OVA developed ~5 times fewer metastatic nodules than control mice and superior systemic protection (~3 times less metastatic nodules) than mice immunized with soluble OVA + CTB (Figure 5B). Therefore, these data demonstrate that in comparison with the soluble antigen, CTB co-administered with a DC-targeted antigen can provide superior systemic immunity against melanoma. Interestingly, antigen specific CD4^+^ T cells could be found in the lungs after i.d. priming, which were slightly increased after DC-targeted vaccination (Supplementary Figure 4C). However, the administration of an anti-CD4 Ab 30 days after priming, and prior to i.v. melanoma challenge, did not affect protection (Figure 5B). Nevertheless, our results show that the immune response induced by a single i.d. dose of CTB co-administered with a DC-targeted antigen provides long-term local and systemic immunity, and, as importantly, the infiltration of CD4^+^ T cells in distal tissues.

### A DC targeted antigen along with CTB induces infiltration of polyfunctional CD4^+^ T cells in the intestine and provides CD4^+^ T cell dependent protection against rotavirus

**Figure 6:**
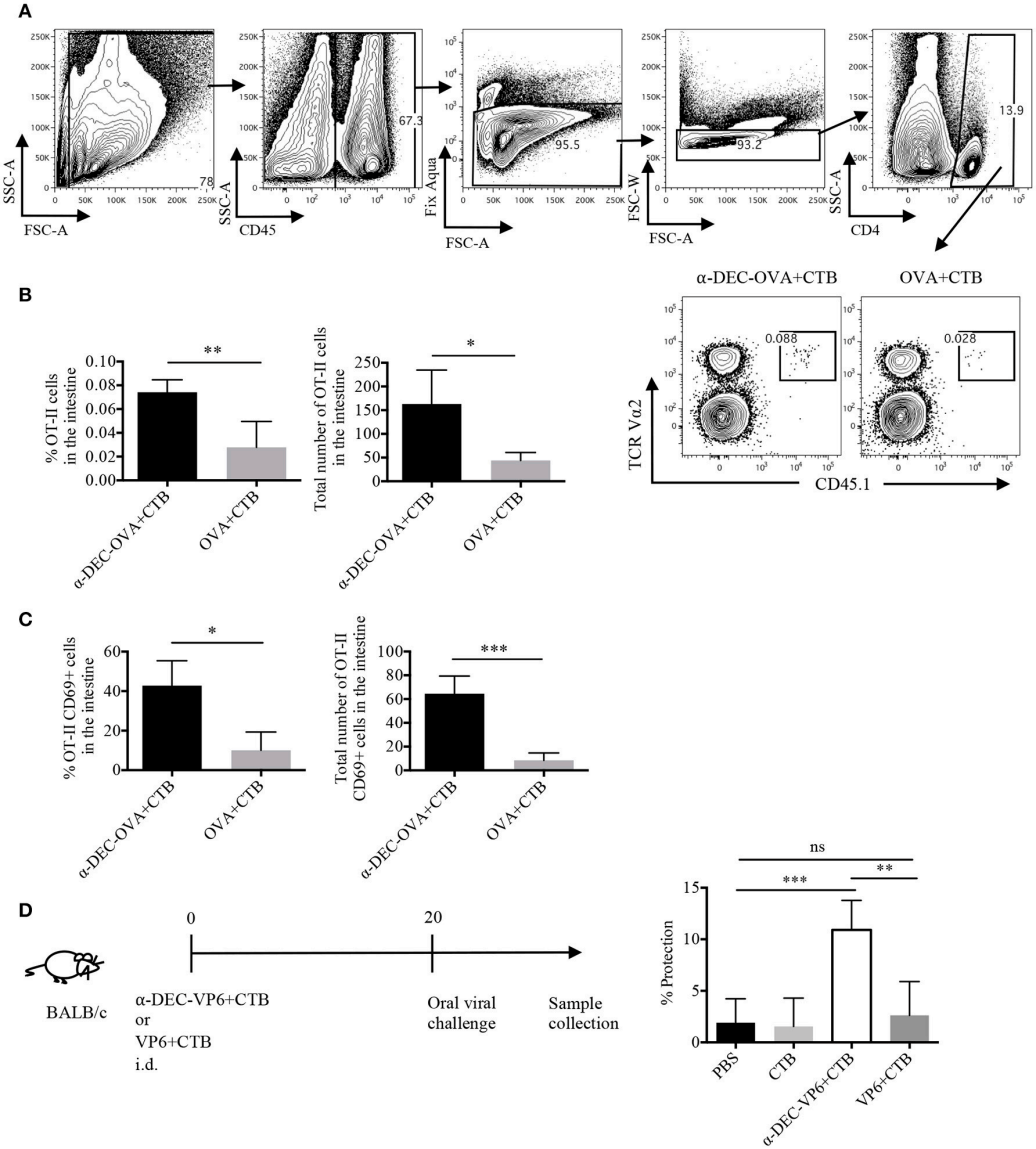
A single dose of a DC-targeted antigen adjuvanted with CTB induces infiltration of antigen specific CD4^+^ T cells in the intestine and partial protection against rotavirus. Mice were treated as in Figure 2 and intestines were collected 7 days post-inoculation. **(A)** OT-II CD45.1^+^ transferred cells were identified as viable CD45^+^CD4^+^TCRVα2^+^CD45.1^+^ cells. **(B)** percentage and total numbers of OT-II CD45.1^+^ cells in the intestines. Mean ± SD, *N* = 5 per group, data pooled from two independent experiments. Unpaired *T*-test (*P < 0.05, **P < 0.005). **(C)** Percentage and total numbers of OT-II CD45.1^+^ cells expressing CD69. Mean ± SD, *N* = 5 per group, data pooled from two independent experiments. Unpaired *T*-test (*P < 0.05, **P < 0.005). **(D)** Diagram showing the immunization strategy followed for viral challenge with murine rotavirus. Stool samples were collected every day up to day 8 and viral load was determined by sandwich ELISA to calculate percentage of protection relative to control (vehicle) mice. Graph depicting percentage of protection after infection. Mean ± SD, *N* = 5 per group, data pooled from two independent experiments. One-way ANOVA with Tukey's multiple comparisons test (ns, *P* > 0.05, **P < 0.005, ***P < 0.0005).

Next, we asked whether the CTB's co-administration with a DC-targeted antigen could induce CD4^+^ T cell responses in another distal tissue, i.e., the intestine. Indeed, very few cells were found in the intestine after i.d. immunization; however, DC-targeted vaccination promoted superior infiltration of OVA-specific CD4^+^ T cells, as compared with the soluble antigen immunization (Figures 6A,B). Furthermore, a higher percentage and number of cells from the intestines of the DC-targeted vaccination group expressed the T RM marker CD69 (Figure 6C; Supplementary Figure 4D).

To evaluate whether i.d. DC-targeted vaccination could provide protection in the intestine we made use of a murine rotavirus model. Rotavirus infection is mostly limited to the small intestine; therefore, the immune response is highly compartmentalized (37). Thus, we made use of a VP6-based vaccine model. VP6 is a highly conserved antigen among different strains of rotavirus (38), and it has been shown to promote protective immunity when targeted to DCs in the presence of Poly IC (30). Furthermore, protection against murine rotavirus, in models of soluble VP6 immunization, is dependent on CD4^+^ T cells (39, 40). Thus, mice were i.d. administered with anti-DEC205-VP6+CTB or soluble VP6+CTB, 20 days before the challenge with oral rotavirus. Only antigen targeting immunization provided intestinal protection (~10%), while soluble immunization did not provide protection against the viral challenge (Figure 6D). Therefore, our results suggest that the immune response elicited by a single dose of i.d. DC-targeted antigen admixed with CTB provides partial long-term immunity in the intestine.

**Figure 7:**
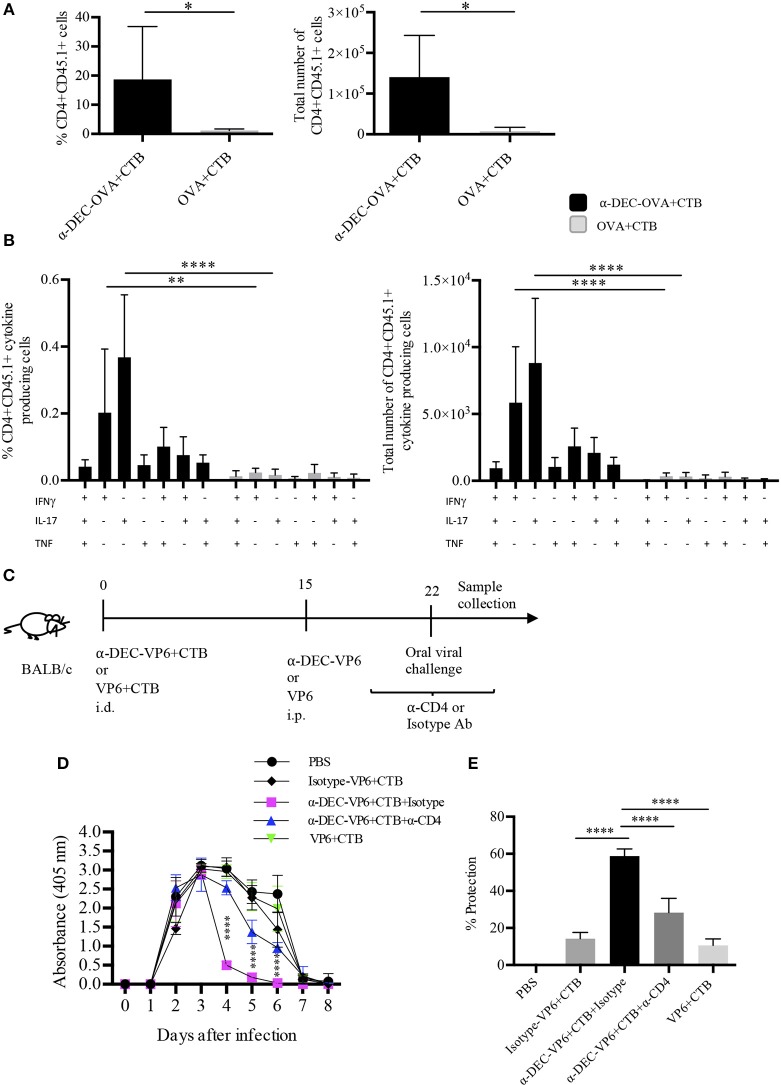
Intradermal prime/i.p. boost immunization with a DC-targeted antigen + CTB induces functional CD4^+^ T cells in the intestine and provides CD4^+^ T cell dependent protection against rotavirus. C57BL6 mice were adoptively transferred with OT-II CD45.1^+^ cells 24 h before i.d. anti-DEC205-OVA or soluble OVA with CTB. Fifteen days later, immune mice received i.p. anti-DEC205-OVA or soluble OVA and after 5 days, mice were sacrificed, and intestines were collected. **(A)** Cells were gated as viable CD45^+^CD4^+^CD45.1^+^ cells to calculate percentage and total number of transferred cells present in the intestine. Mean ± SD, *N* = 6 per group, data pooled from two independent experiments. Unpaired *T*-test (*P < 0.05). **(B)** Freshly isolated cells were stimulated 4 h with cell cocktail stimulation + protein transport inhibitor. Graphs of percentage and total numbers of CD4^+^CD45.1^+^ cytokine producing cells (gated as in **B**). Boolean combinations were calculated using FlowJo software. Mean ± SD, *N* = 6 per group, data pooled from two independent experiments. Two-way ANOVA with Bonferroni's multiple comparison test (**P = 0.0017, ****P < 0.0001). **(C)** Strategy followed for oral viral challenge with murine rotavirus after i.d. immunizations and i.p. boost. Mice immunized with anti-DEC-VP6+CTB received i.p. anti-CD4 or the control isotype Ab, before, during and after the viral challenge. **(D)** Stool samples were collected every day up to day 8 and viral load was determined by sandwich ELISA. **(E)** Percentage of protection relative to control (vehicle) mice, calculated as area under the curve (From **D**). Mean ± SD, *N* = 5–8 per group, data pooled from two independent experiments. Two-way ANOVA with Tukey's multiple comparisons test.

The development of partial protection after a single i.d. dose of a DC-targeted antigen could have been due to poor infiltration of functional T cells in the intestine. Therefore, we asked whether a prime/boost immunization scheme could expand the specific CD4^+^ T cells. To answer this question, mice were i.d. immunized with a DC-targeted antigen or a soluble antigen admixed with CTB. Fifteen days later, mice received, i.p. the targeted or soluble antigen. After 5 days, we observed a greater expansion of antigen-specific CD4^+^ T cells in the intestine after DC-targeted prime/boost, compared to the soluble antigen prime/boost group (Figure 7A). In addition, DC-targeted prime/boost promoted the expansion of IL-17^+^ CD4^+^ T cells, that can also produce other cytokines like IFNγ and/or TNFα, in contrast to soluble antigen immunization (Figure 7B).

The above results prompted us to discern whether the prime/boost immunization strategy could improve protection in the murine rotavirus model. To this end, mice received anti-DEC205-VP6 or soluble VP6 admixed with CTB, via the i.d. route; 15 days later they received i.p. anti-DEC-VP6 or VP6 only. After 7 days, mice were orally challenged with rotavirus (Figure 7C). Four days after the challenge, the viral load dramatically dropped in stool samples from the DC-targeted vaccination group (Figure 7D). This meant ~60% protection against infection relative to naïve mice (Figure 7E). Protection relied on the antigen being targeted to DCs, since the isotype Ab conjugated with VP6 and admixed with CTB only provided partial protection (~15%). Protection was significantly dampened when CD4^+^ T cells were depleted by the administration of anti-CD4 antibody. On the other hand, soluble antigen vaccination provided only partial protection against infection (~15%; Figures 7D,E).

Collectively, our results show that i.d. administration of DC-targeted antigens admixed with CTB promotes the infiltration of polyfunctional CD4^+^ T cells in the intestine. It is important to point out that our data suggest that this response provides long-term immunity against a pathogen whose clearance is partially dependent on CD4^+^ T cells.

## Discussion

Immunization strategies that confer broad long-lasting immunity mediated by CD4^+^ T cells are fundamental to eradicate modern pandemics. To achieve this goal, mAbs targeting antigen to DEC205^+^ DCs, in combination with maturation stimuli, is one of the most promising strategies. Here we have demonstrated that DC-targeted antigens admixed with CTB promote the development of long-lasting systemic protective CD4^+^ T cell responses.

Successful DC-targeted vaccination requires DC stimulation by strong adjuvants, which ultimately promotes T cell responses. Therefore, we studied the activation and accumulation of DCs following the CTB's i.d. administration. It took 72 h to observe both DC activation and accumulation in the skin; in contrast, other adjuvants (i.e., LPS, CpG, flagellin and the complete cholera toxin) can induce local activation as soon as 6 to 24 h (9, 41–45). Differences could be related to the receptors engaged by CTB on the DCs (17, 18). The late activation of skin DCs was also seen in the SDLN, where activated migratory DCs accumulated 72 h after CTB inoculation. These findings could explain why others have failed at demonstrating activation and accumulation of DCs in draining lymph nodes 2–24 h following CTB administration (44, 46). Therefore, while other adjuvants can promote rapid activation and accumulation of DCs, our results indicate that CTB induces late activation and accumulation of skin DCs.

Interestingly, the accumulation and the activated phenotype of DCs were still observed after 7 days, in both the skin and the SDLN. Similar observations have been reported after the administration of CpG, alum or the MF59 oil-in-water emulsion (47), which induced accumulation in the muscle of MHC-II^+^ cells up to 4 days after inoculation. The same phenomenon was true for resident lymph node DCs following CTB administration. These findings suggest that CTB can stimulate various populations of DCs for a prolonged time, which could potentially lead to sustained and diverse DC-T cell interactions. Noticeably, the late accumulation of activated skin and lymph node-DCs correlated with the priming of CD4^+^ T cell responses observed at day 7, following the CTB's co-administration with antigen. Together, our findings shed light on the CTB's controversial ability to activate DCs *in vivo*.

Antigen targeting to DEC205^+^ DCs is a promising system to promote CD4^+^ T cell responses (4, 6). Indeed, i.d administration of anti-DEC205-OVA increased the efficiency of antigen presentation relative to the soluble OVA. It was not, however, as large as reported by previous publications that used the s.c. or i.p. routes. Because the SDLN are very close to the inoculation site, i.d. administration of very small quantities of soluble antigen can efficiently promote CD4^+^ T cell proliferation, in contrast with the s.c or i.p. routes (27). Furthermore, i.d. administration of anti-DEC205-OVA clearly induced a different activation of CD4^+^ T cells as compared with soluble OVA. Not only did it induce cells to proliferate more, but it also induced a marked downregulation of CD69, which is necessary for T cells' egress to the periphery (31, 32). In this regard, the soluble antigen along with CTB promoted a localized CD4^+^ T cell response, while a DC-targeted antigen admixed with CTB induced systemic CD4^+^ T cell responses. Considering that we did not observe DC activation in distal sites, our results suggest that following i.d. DC-targeted vaccination; the priming occurs in the SDLN, and then, activated CD4^+^ T cells migrate to infiltrate the site of inoculation and, remarkably, other peripheral tissues. Therefore, our results suggest that the priming induced by a DC-targeted antigen admixed with CTB promotes unique systemic CD4^+^ T cell responses.

Indeed, a DC-targeted antigen along with CTB induced a combined and systemic Th1/Th17 response. In contrast, soluble antigen immunization promoted a skewed localized Th1 response, which is similar to that observed when using CTB as an adjuvant linked with antigens or admixed with pathogen derived antigens (22–24, 26). Furthermore, the CTB's combination with a DC-targeted antigen promoted the differentiation of polyfunctional Th cells. It has been documented that anti-DEC205-antigen Abs admixed with experimental adjuvants—i.e., CpG oligonucleotides, flagellin (9) and Poly IC (7)—induce differentiation of polyfunctional CD4^+^ T cells that produce IFNγ, TNFα, and IL2. However, none of these adjuvants are able to induce Th17 differentiation (48–50). In our model, the superior induction of Th17 cells seemed to depend on both the adjuvant and the antigen being directly delivered to DEC205^+^ DCs, since soluble antigen vaccination induced IL-17^+^ antigen specific CD4^+^ T cells only marginally. To our knowledge, this is the first report showing induction of systemic polyfunctional CD4^+^ T cell responses that include IL-17^+^ cells after antigen targeting to DEC205^+^ DCs by genetically engineered mAbs admixed with CTB.

We also demonstrate that a DC-targeted antigen admixed with CTB efficiently promotes the generation of memory CD4^+^ T cells, something that has not been extensively explored after performing DC-targeted vaccination. Here, using cell surface markers, we found in the SDLN the presence of circulating and re-circulating memory CD4^+^ T cells after using a DC-targeted antigen or a soluble antigen admixed with CTB. Strikingly, the high infiltration of CD4^+^ T cells after soluble antigen immunization did not translate into more T RM differentiation. In contrast, DC-targeted vaccination induced superior differentiation of CD4^+^ T RM cells at the site of inoculation and, more importantly, at a distal nonlymphoid tissue, i.e., the intestine. This is similar to what has been observed in studies inoculating recombinant vaccinia virus expressing OVA through skin scarification, which induces the differentiation of protective T RM cells in the skin and lungs (51–53). Thus far, there are only a couple of publications reporting CD8^+^ T RM cell differentiation after immunization with anti-DEC205-antigen mAbs, using LPS (54) or Poly IC (55) as adjuvants. However, none of them have shown the presence of T RM cells in distal sites after local vaccination. Our observations suggest that antigen targeting to DEC205^+^ DCs, in combination with CTB, is an effective strategy to promote systemic differentiation of CD4^+^ T RM cells. This is of particular relevance in light of recent studies, pointing to T RM cells as essential players against several infections (34, 36, 56) and melanoma (53, 55) protection.

Following this line, DC-targeted and soluble antigen vaccination provided similar long-term protection against subcutaneous melanoma. This could be related to the protective capacity of both circulating and T RM cells against melanoma (53). However, we found that DC-targeted vaccination provided superior systemic protection against pulmonary tumor growth. Although we found antigen specific CD4^+^ T cells in the lungs of immune mice, the administration of a neutralizing anti-CD4 Ab during the memory phase did not abrogate protection against i.v. melanoma. This is contrary to melanoma studies in CD4 knockout mice, where protection is partially dampened (4). Therefore, we cannot completely rule out the participation of CD4^+^ T cells in the priming of protective CD8^+^ T cell responses against i.v. melanoma. In this regard, our results suggest that protection in the lungs could be primarily mediated by memory CD8^+^ T cells after DC targeted vaccination using CTB as adjuvant. This idea is supported by the fact that CD8^+^ T cells are efficiently activated by anti-DEC205 Abs (4, 6) and by antigens linked to CTB (57, 58). Since priming occurred in the SDLN, our findings suggest that DC-targeted vaccination using CTB as adjuvant can be used as an efficient immunization strategy to provide systemic long-term immunity against melanoma.

Interestingly, DC-targeted vaccination-induced systemic CD4^+^ T cell responses translated into protection in the intestine. This could have been mediated by the T RM and polyfunctional CD4^+^ T cells found in the intestine after DC-targeted vaccination. However, a single immunization induced only small numbers of T cells in the intestine and partial protection. Since protective immunity correlates with high numbers of functional cells infiltrating the site of infection, we took advantage of the ability of anti-DEC205 Abs to disseminate systemically (6) to successfully expand the antigen specific CD4^+^ T cells in the intestine through a DC-targeted antigen + CTB i.d. prime/DC-targeted antigen i.p. boost. Remarkably, this strategy promoted higher numbers of IL-17^+^ CD4^+^ T cells to be present in the intestine, as well as polyfunctional CD4^+^ T cells. Furthermore, the prime/boost scheme dramatically improved protection against the oral viral challenge, but only when the antigen was targeted to DCs. Moreover, the protection observed was superior than the one reported by s.c. administration of the same antibody in the presence of Poly IC, which was related to the development of Th1 responses (30). Also, protection in our model was partially dependent on CD4^+^ T cells, according with the CD4 blockade experiments. However, we cannot exclude the participation of CD8^+^ T cells. These findings indicate that DC-targeted antigens admixed with CTB promote infiltration of the intestine with functional CD4^+^ T cells capable of mediating protection against pathogens with intestine tropism.

Our results extend the advantages of immunization with antigens targeted to DEC205^+^ DCs with mAbs in combination with strong adjuvants (CTB) to induce high quality systemic immune responses that translate into protection. We propose that a DC-targeted antigen can be co-administered with CTB i.d.; a suitable novel combination with potential human use, for the generation of protective, systemic and long-lasting Th17 CD4^+^ and polyfunctional responses, which, importantly, are characterized by CD4^+^ T RM cells. Furthermore, this immunization strategy could be used to fight infections and tumors.

## Ethics statement

This study was carried out following the recommendations of the Institutional Ethics Committee and the Comité Local de Investigación en Salud, Protocol number R-2015-785-023. All procedures for animals were approved by the Animal Ethics Committee of the Faculty of Medicine at UNAM, and they followed the Mexican Official Guide (NOM 062-ZOO-1999) for the care and use of laboratory animals.

## Author contributions

LA-H performed the majority of the experiments, interpreted the data and drafted the manuscript. OB-G performed the murine rotavirus protection assay. OM-C processed intestine samples. AT-S helped with skin DCs analysis. AT-S and AG-L performed confocal microscopy experiments. FE-G and LG-X contributed to design the murine rotavirus experiments. GS provided transgenic OT-II CD45.1^+^ mice and helped with the interpretation of data. JI helped to train the first author in experimental techniques, provided reagents necessary for the study (i.e., anti-DEC-OVA mAb), helped with the design of the study and the interpretation of results, and revised the manuscript. LB conceived and directed the project and revised the manuscript. All the authors reviewed the manuscript critically.

### Conflict of interest statement

The authors declare that the research was conducted in the absence of any commercial or financial relationships that could be construed as a potential conflict of interest. The reviewer LF and handling Editor declared their shared affiliation.

## Abbreviations

CTB: cholera B subunit
i.d.: intradermal
i.v.: intravenous
s.c.: subcutaneous
DC: dendritic cell
Ab: antibody
mAb: monoclonal antibody
SDLN: skin draining lymph node
T RM: resident memory T cell
T CM: central memory T cell
T EM: effector memory T cell.
