# Supplementary material for: The Nontoxic Cholera B Subunit Is a Potent Adjuvant for Intradermal DC-Targeted Vaccination
Source: Front Immunol. 2018 Sep 27;9:2212. doi: 10.3389/fimmu.2018.02212 (PMC6171476; doi:10.3389/fimmu.2018.02212)
Supplement: Supplementary file 1 [file Data_Sheet_1.PDF]

Supplementary Material

THE NONTOXIC CHOLERA B SUBUNIT IS A POTENT ADJUVANT FOR INTRADERMAL DC-TARGETED VACCINATION

Laura Antonio-Herrera[1, 2], Oscar Badillo-Godinez[3], Oscar Medina-Contreras[4], Araceli Tepale-Segura[1] , Alberto García-Lozano[1], Lourdes Gutierrez-Xicotencatl[3], Gloria Soldevila[5], Fernando Esquivel-Guadarrama[6], Juliana Idoyaga[7*], Laura C. Bonifaz[1*]

[1] Unidad de Investigación Médica en Inmunoquímica, Hospital de Especialidades, Centro Médico Nacional Siglo XXI, Instituto Mexicano del Seguro Social, Mexico City, Mexico.

[2] Universidad Nacional Autónoma de México (UNAM), Mexico City, Mexico.

[3] Centro de Investigación Sobre Enfermedades Infecciosas, Intituto Nacional de Salud Pública, SSA, Cuernavaca, Morelos, Mexico.

[4]Immunology and Proteomics Laboratory, Mexico Children´s Hospital "Federico Gómez", Mexico City, Mexico

[5] Departamento de Inmunología, Instituto de Investigaciones Biomédicas, UNAM, Mexico City, Mexico.

[6] Laboratorio de Inmunología Viral, Facultad de Medicina, UAEM, Cuernavaca, Morelos, Mexico.

[7] Department of Microbiology and Immunology, Stanford University School of Medicine, Stanford, CA, USA.

*** Correspondence:**
Dr. Laura C. Bonifaz
labonifaz@yahoo.com

Dr. Juliana Idoyaga

jidoyaga@stanford.edu

Supplementary Figures

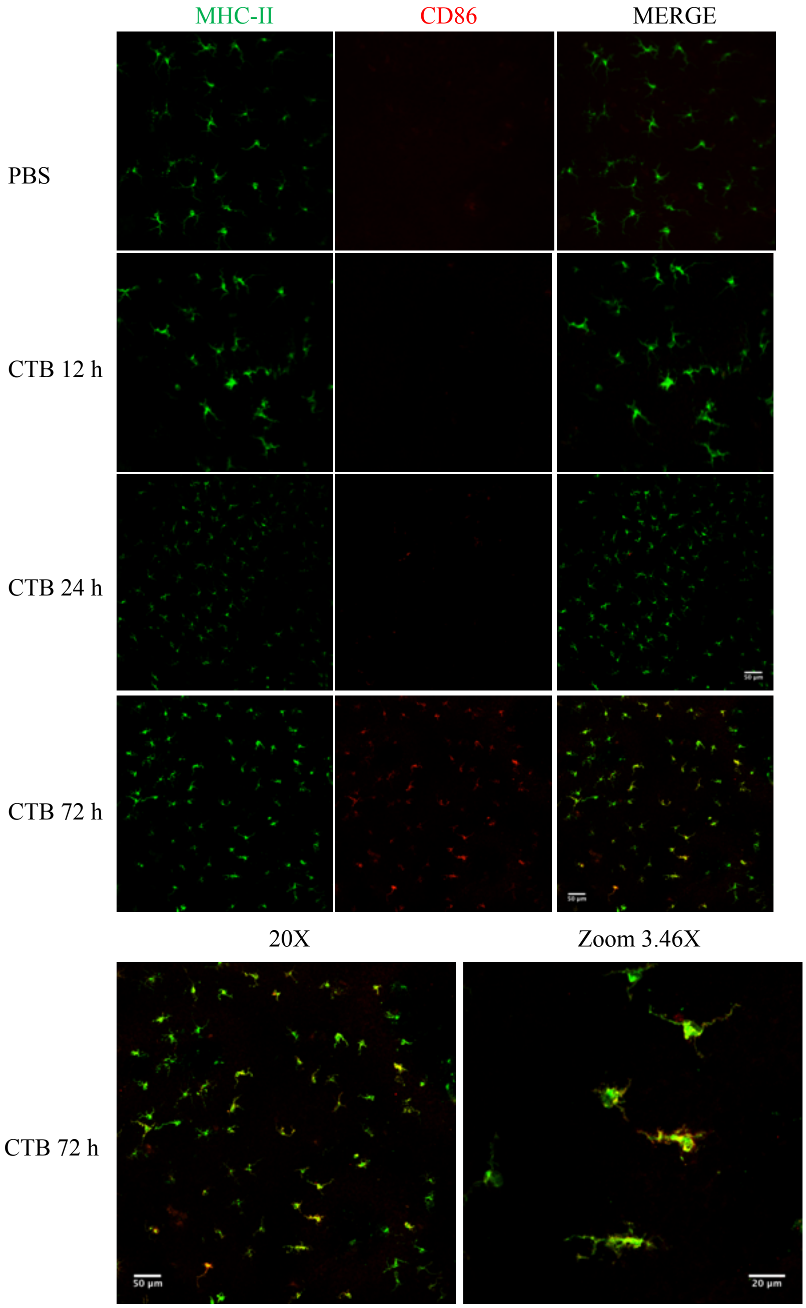

Supplementary Figure 1. GFP-MHC-II mice received i.d. in both ears 10 µg of CTB or PBS. After 12, 24 or 72 hours, epidermal sheets were obtained, stained with anti-CD86-PE and mounted for confocal microscopy. MHC-II: Green, CD86: Red.

A

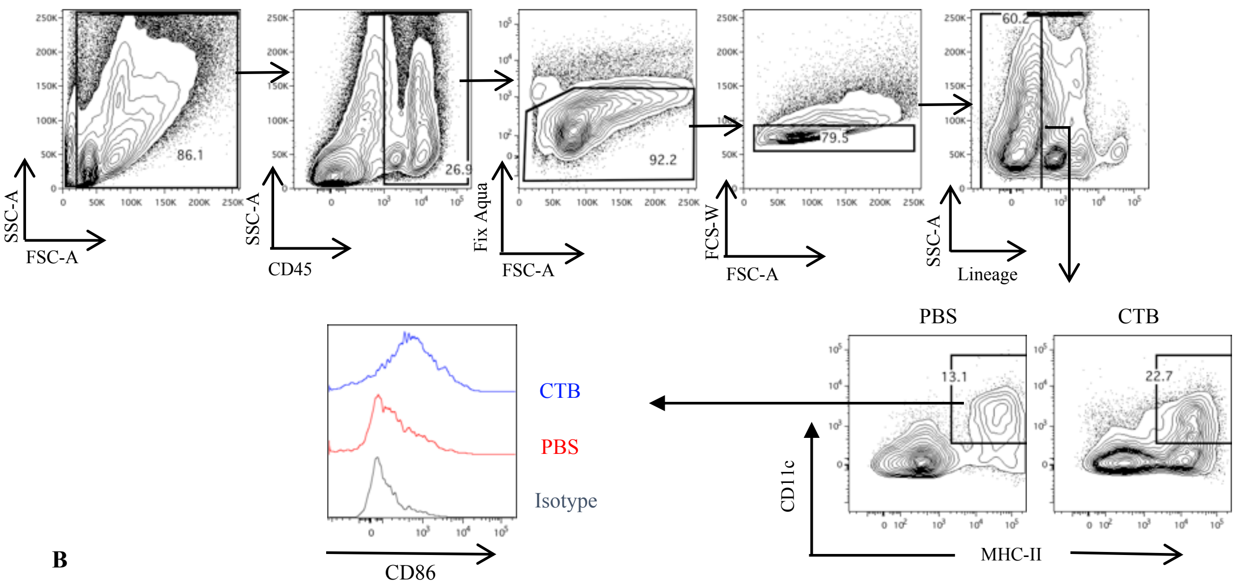
SSC-A
FSC-A
86.1
CD45
26.9
Fix Aqua
92.2
FCS-W
79.5
Lineage
60.2
PBS
CTB
13.1
22.7
CD11c
MHC-II
CTB
PBS
Isotype
CD86

B

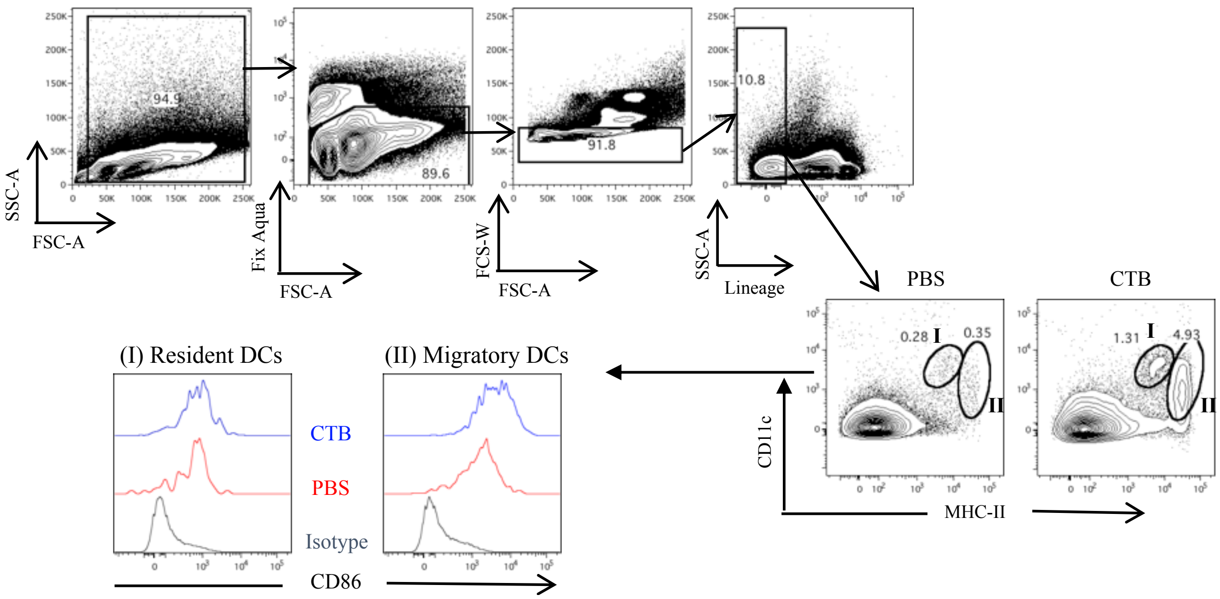
SSC-A
FSC-A
94.9
Fix Aqua
89.6
FCS-W
91.8
Lineage
10.8
PBS
CTB
0.28
I
0.35
II
1.31
4.93
CD11c
MHC-II
(I) Resident DCs
(II) Migratory DCs
CTB
PBS
Isotype
CD86

C

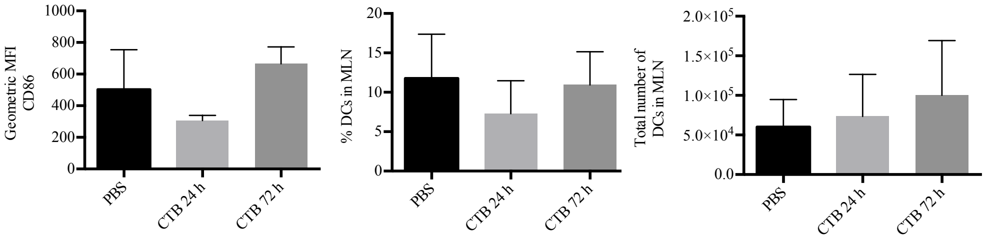
Geometric MFI CD86
% DCs in MLN
Total number of DCs in MLN
PBS
CTB 24 h
CTB 72 h

Supplementary Figure 2. C57BL6 mice received i.d. in both ears 10 µg of CTB or PBS. After 24, 72 hours or seven days, mice were sacrificed to collect the skin and SDLN. (A) Gating strategy followed to identify DCs in the skin. A representative contour plot is shown of DCs and histogram of CD86 expression after 72 h. (B) Gating strategy followed to identify DCs in the SDLN. Cells were divided as (I) CD11c+MHC-II low resident DCs or (II) CD11c+MHC-II high migratory DCs. A representative contour plot is shown of DCs and histogram of CD86 expression after 72 h. (C). The mesenteric lymph nodes of mice were also obtained to evaluate the presence of DCs and C86 expression. CD11c+MHC-II+ DCs were gated as in B. Mean ± SD, N=4 per group, data pooled from two independent experiments.

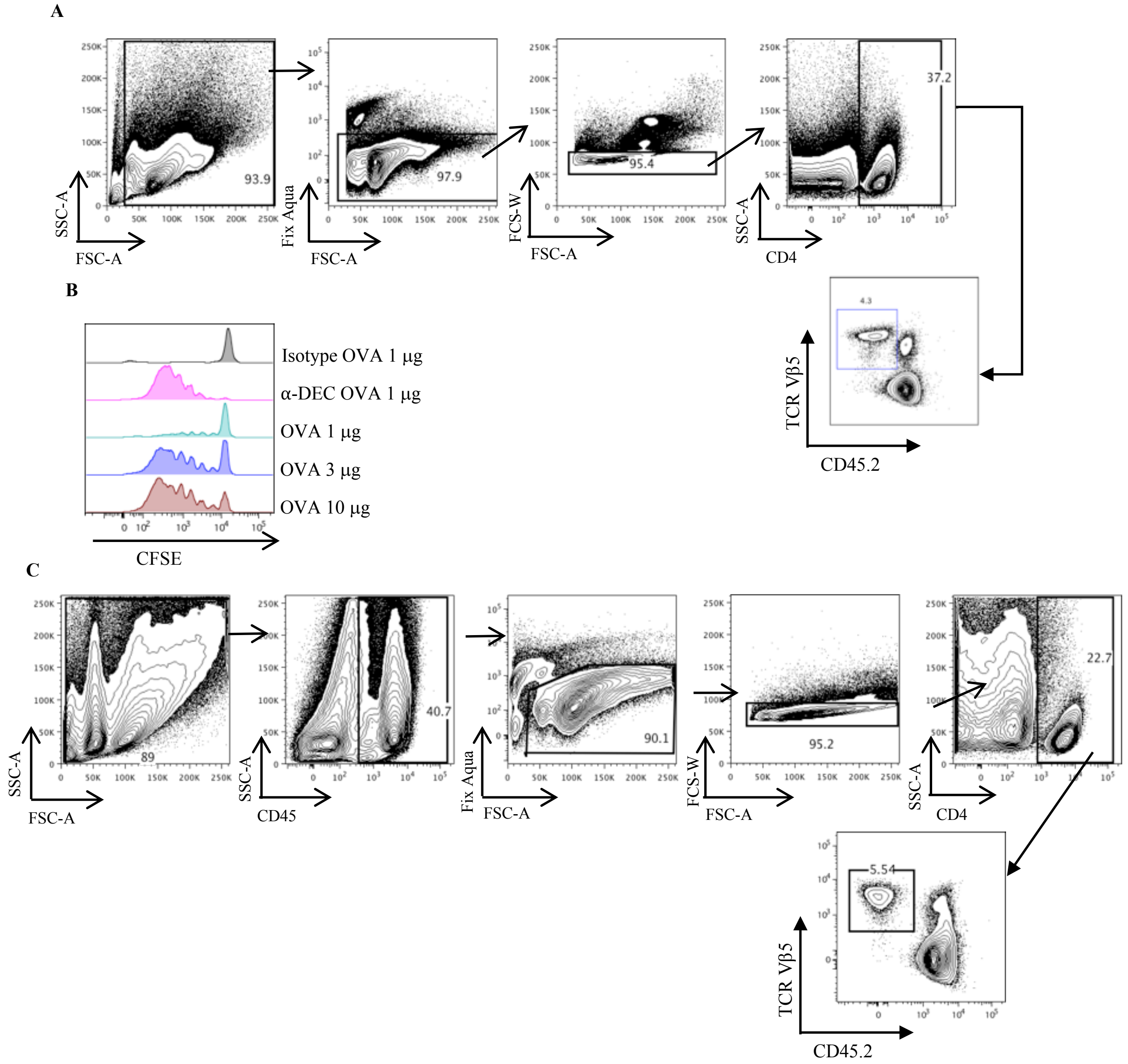

Supplementary Figure 3. C57BL6 mice were adoptively transferred with CFSE-labeled OT-II CD45.1+ cells, 24 h later i.d. immunized in both ears as indicated and three days after sacrificed for

SDLN harvesting. (A) Gating strategy followed to identify OT-II CD45.1+ cells in the SDLN. (B) CFSE dilution by OT-II CD45.1+ cells. (C) Gating strategy followed to identify OT-II CD45.1+ cells in the skin.

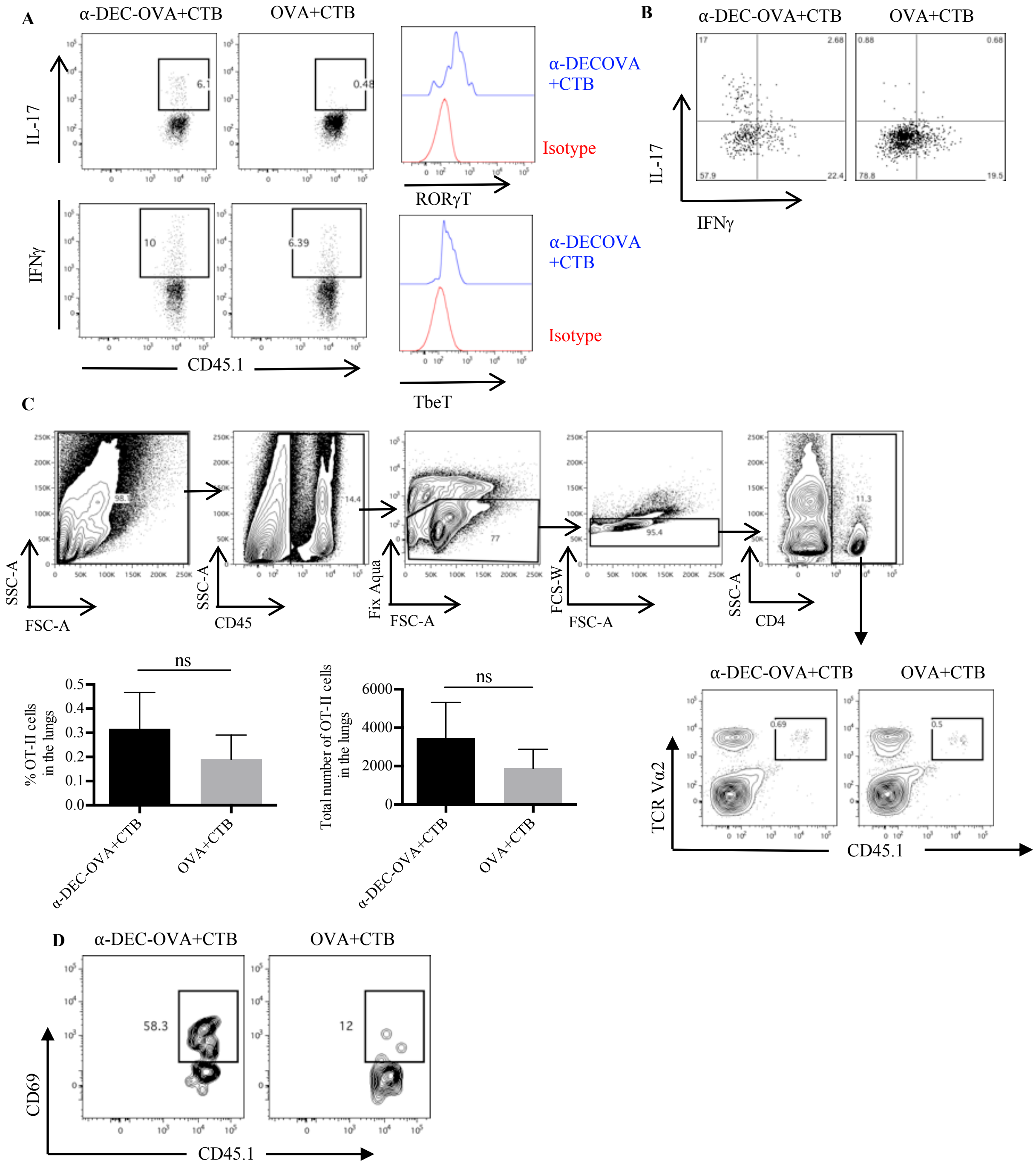

Supplementary Figure 4. C57BL6 mice were adoptively transferred with OT-II CD45.1+ cells, 24 h later i.d. immunized in both ears as indicated and seven days after sacrificed for SDLN, skin and lung harvesting. Cells from the SDLN were incubated with OVA 223-339 for 48 h followed by cell stimulation with cell cocktail stimulation + protein transport inhibitor for 4 h. (A) Representative dot

plots of IFNγ and IL-17 intracellular expression and histograms showing the expression of RORγT and T-bet. (B) Cells from the skin were incubated 4 h with cell cocktail stimulation + protein transport inhibitor. Representative plots of IFNγ and IL-17 intracellular expression. (C) Gating strategy followed to identify OT-II CD45.1 cells in the lungs. A representative contour plot is shown. Graphs of percentage and total number of OTI-II CD45.1+ cells in the lungs. Mean ± SD, N=5 per group, data pooled from two independent experiments. Unpaired T-test (ns, P > 0.05). (D) CD69 expression by OT-II CD45.1+ cells from the intestine.

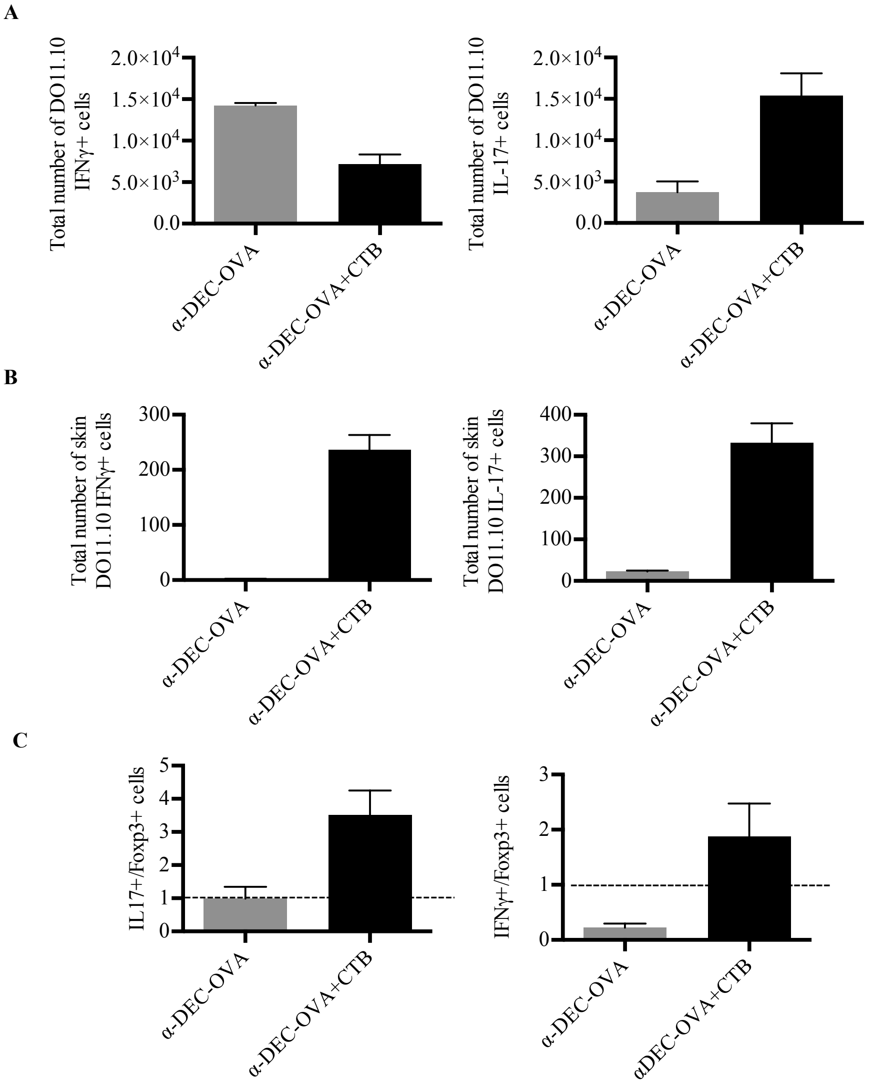

Supplementary Figure 5. BALB/c mice were adoptively transferred with DO11.10 Thy1.1+ cells, 24 h latter i.d. immunized in both ears, as indicated, and sacrificed at the indicated time points for SDLN or skin harvesting. (A) Total number of IFNγ+ and IL-17+ DO11.10 Thy1.1+ cells after incubation of SDLN cells, obtained three days post-immunization, with stimulation + protein transport inhibitor for 4 h. (B) Total numbers of IFNγ+ and IL-17+ DO11.10 Thy1.1+ cells after *in vitro* re-stimulation as in

(A) of skin cells obtained three days post-immunization. (C) At day 14, skin cells were either stained for Foxp3 or stimulated as in (A) and cytokine/Foxp3+ ratio of DO11.10 Thy1.1+ cells was calculated.
